# Prevalence of Vitamin D Deficiency during Second Trimester of Pregnancy in Shanghai China, Risk Factors and Effects on Pregnancy Outcomes

**Published:** 2018-08

**Authors:** Ling YANG, Lige SONG, Xiao XU, Yihan LIU, Huijuan LI, LongYing TANG

**Affiliations:** 1. Dept. of Obstetrics, Changning Maternity and Infant Health Hospital, Shanghai, China; 2. Dept. of Endocrinology, Tongji Hospital, School of Medicine, Tongji University, Shanghai, China

**Keywords:** Vitamin D deficiency, Second trimester of pregnancy, Prevalence, Risk factor, Pregnant outcome

## Abstract

**Background:**

Vitamin D plays important roles in various physiological processes. Vitamin D deficiency is common among pregnant women in some regions, such as China. Our study aimed to determine the prevalence of Vitamin D deficiency during second trimester of pregnancy in Shanghai China, and explore its risk factors and effects on pregnant outcomes.

**Methods:**

Overall, 23100 pregnant women (2013 to 2017, Shanghai, China) were included and vitamin D concentrations were measured at 16 weeks of gestation. Correlations between vitamin D concentrations and participants’ general data and maternal and infant outcomes were analyzed by chi square test. Non-conditional multivariate logistic regression analysis was used to screen the independent risk factors for vitamin D deficiency.

**Results:**

Vitamin D deficiency was significantly correlated with aging, education level, smoking, dirking, BMI before pregnancy, body weight gain during pregnancy (*P*<0.01), the use of vitamin D supplement and milk consumption, and older than 30 years, drinking, smoking, BMI before pregnancy> 36, body weight gain during pregnancy< 40g per day, no daily milk consumption, no vitamin D supplement, and education lever below college were independent risk factors for vitamin D deficiency in second trimester of pregnancy. In addition, vitamin D deficiency in second trimester of pregnancy was closely correlated with the occurrence of a serious of adverse maternal and infant outcomes.

**Conclusion:**

Vitamin D deficiency was still common among women in second trimester of pregnancy in Shanghai China. Vitamin D deficiency was closely correlated with the occurrence of a serious of adverse maternal and infant outcomes.

## Introduction

Vitamin D deficiency is a widespread public health problem worldwide. In China, the rapid economic development and the shift of diet behaviors to Western style in past several decades significantly increased the prevalence of many types of chronic diseases, including vitamin D deficiency (1, 2). Vitamin D deficiency is considered to be common in pregnant women (3, 4), and is closely correlated with the a serious of serious short- and long-term health problems in offspring, including skeletal problems, impaired growth, asthma type 1 diabetes, schizophrenia and so on (5). Therefore, prevention of vitamin D deficiency is a key in improving maternal and infant outcomes.

Problems with urbanization, such as air pollution, less physical activity and increased sedentary occupations have been proved to be at least partially responsible for the increased prevalence of vitamin D deficiency in developed countries and areas (6). Besides that, increased incidence of obesity in recently years may also a major cause of high increased prevalence of vitamin D due to the dependency of vitamin D status on body mass index (BMI) (7). Shanghai is one of the most developed areas in China, and a considerable proportion of females in this area were occupied by sedentary jobs, which inevitably increases the incidence of vitamin D deficiency and adverse pregnancy events such as miscarriage (8). Vitamin D status of residents in Shanghai has been reported by a previous study (9). However, it has been 5 years since this report, and the effects of vitamin D deficiency on maternal and infant outcomes, especially in the second trimester of gestation were not reported.

We aimed to update the information of the prevalence of vitamin D deficiency during second trimester of pregnancy in Shanghai, China, and to explore its risk factors of vitamin D deficiency during second trimester and its effects on pregnant outcomes.

## Materials and Methods

### Subjects

A total of 23100 pregnant women were recruited from January 2013 to October 2017 (in winter timer) in Shanghai Changning District Maternal and Child Health Hospital, Shanghai, China. The age of those patients ranged from 22 to 44 yr, with an average age of 32±4.2 years. Manual workers and the ones with kidney disease, chronic liver disease, severe cardiocerebral vascular disease, or cancer were excluded. Patients with a history of diabetes mellitus were also not included.

This study was approved by the Ethics Committee of Shanghai Changning District Maternal and Child Health Hospital. All participants signed informed consent.

### Measurement of blood 25OHD and grouping

Blood was extracted from each participant by venipuncture at 16 weeks of gestation. OCTEIA 25-Hydroxy vitamin D kit (Immunodiagnostic Systems Linited, UK) was used to determine serum 25OHD concentration. Patients were divided into different groups according to Institute of Medicine (IOM) cut-offs: serum 25(OH)D <30 nmol/L, deficiency; between 30–50 nmol/L, inadequacy; and >50 nmol/L, sufficiency (9).

### Data collection

Participants were interviewed face to face to obtain their basic formations. Smoking habits were grouped into “none” (never smoking), “light” (nor more 70 cigarettes/week), and “heavy” (70 cigarettes or more per week). Alcohol intake were classified into “none” (never drinking during past 1 year), “light” (drinking sporadically), and “heavy” (drinking every more than 3 days a well). Other information, such as age, education level, the use of vitamin D supplement and consumption of milk products were also collected. BMI was calculated and body weight gain during pregnancy was recorded.

### Determination of maternal and infant outcomes

Gestational hypertension was defined as new onset hypertension (ystolic blood pressure (BP) ≥ 140 mmHg and/or diastolic BP >=90 mmHg measured twice 4 hours apart) at more than 20 weeks of gestation. Diagnostic criteria for gestational diabetes mellitus: normal 75gOGTT; fasting blood glucose <5.1mmol/L; blood glucose at 1h after glucose uptake (75g) <10.0 mmol/L; blood glucose at 2 h after glucose uptake (75g) <8.5 mmol/L, patients with any abnormal index were diagnosed as gestational diabetes mellitus. Pre-eclampsia was defined as new onset hypertension after 20 weeks of gestation combined with proteinuria (>=0.3 g/day or >=2+ dip-stick), or haemolysis, or eclampsia, low platelets, and elevated liver enzymes (HELLP syndrome), or new onset of proteinuria, on the background of essential hypertension. Adverse placental outcomes were determined and included preeclampsia, gestational hypertension, intrauterine fetal death, placental abruption and so on.

### Statistical analysis

SPSS 17.0 statistical software (Chicago, IL, USA) was used for all statistical analysis. Measurement data were expressed by *x̅* ± *s* and compared by *t* test. Count data were by χ^2^ test. Indicators with statistical significance among vitamin D deficiency group, inadequacy group and sufficiency group were assigned values and set as independent variables. Age<= 30 yr, 0; Age>30, 1; education level >college, 0; education level <college, 1; consume milk products daily, 0; not consume milk products daily, 1; for smoking and drinking habit, none, 0; light, 1; heavy 2; BMI <=26, 0; BMI >26, 1; body weight gain >40g/day, 0; body weight gain <40 g/day, 1. Non-conditional multivariate logistic regression analysis was used to screen the independent risk factors for vitamin D deficiency.

## Results

### Correlations between vitamin D status and general data of pregnant women

Overall, 23100 pregnant women were included. Among them, 7526 females showed vitamin D deficiency, accounting for 32.58%, and 7649 females showed vitamin D inadequacy, accounting for 33.11%. As shown in Table 1, vitamin D status was significantly correlated with age, education level, smoking habit, drinking habit, BMI, body weight gain, the use of vitamin D supplement and consumption of milk (*P*<0.01). However, vitamin D status was not closely correlated with race.

**Table 1:** Correlations between vitamin D status and general data of pregnant women

***Items***	***Cases***	***Deficiency***	***Inadequacy***	***Sufficiency***	***χ^2^***	***P value***
Age(yr)						
<=30	14772	4728	4875	5169	9.886	0.007
>30	8328	2798	2774	2756		
Race						
Han	20710	6737	6884	7089	1.485	0.476
Minor	2390	789	765	836		
BMI before pregnancy						
>26	3483	1455	1201	827	237.157	p<.001
<=26	19617	6071	6448	7098		
Body weight gain during pregnancy						
>=<40g per day	3142	1337	1127	678	312.883	p<.001
<40g per day	19958	6189	6522	7247		
College education						
Yes	10491	3219	3181	4091	201.478	p<.001
No	12609	4307	4468	3834		
Smoking status						
None	18733	5795	5935	7003	516.165	p<.001
Light	3057	1129	1140	788		
Heavy	1310	602	574	134		
Drinking status						
None	15772	4882	5014	5876	193.357	p<.001
Light	4462	1621	1610	1231		
Heavy	2866	1023	1025	818		
Vitamin D supplement						
Yes	7482	2350	2410	2722	21.245	p<.001
No	15618	5176	5239	5203		
Consume milk products daily						
Yes	10977	3519	3581	3877	24.976	p<.001
No	12123	4007	4068	3834		

### Analysis of risk factors for vitamin D deficiency

Non-conditional multivariate logistic regression analysis was used to identify independent risk factors for vitamin D deficiency. Older than 30 years, drinking, BMI before pregnancy >26, body weight gain during pregnancy<40g/day, smoking, none-daily milk consumption, none-vitamin D supplement, and education lever below college were independent risk factors for vitamin D deficiency (Table 2) (*P*<0.05).

**Table 2:** Analysis of risk factors for vitamin D deficiency

***Factors***	***Regression coefficient***	***Standard error***	***Wald value***	***Odd ratio***	***95% confidence interval***	***P value***
Aging	0.492	0.064	5.88	1.371	1.234~1.713	0.021
Education level below college	0.32	0.093	11.57	1.387	1.401~1.616	0.011
BMI before pregnancy >26	0.387	0.087	10.167	1.306	1.334~1.698	0.007
Body weight gain during pregnancy <40g per day	0.333	0.092	11.022	1.282	1.277~1.631	0.009
Smoking	0.274	0.091	12.34	1.401	1.356~1.701	0.009
Drinking	0.314	0.095	12.12	1.36	1.445~1.696	0.008
No Vitamin D supplement	0.413	0.072	8.64	1.382	1.245~1.721	0.012
Not consume milk products daily	0.331	0.097	12.21	1.45	1.343~1.682	0.017

### Correlations between vitamin D deficiency and adverse maternal and infant outcomes

Adverse maternal and infant outcomes, including preterm birth, adverse placental outcome, preeclampsia, gestational diabetes mellitus, gestational hypertension, placental abruption and intrauterine fetal death were recorded during follow-up. As shown in Table 3, vitamin D was significantly correlated with the occurrence of pre-term birth, gestational diabetes mellitus, adverse placental outcome, pre-eclampsia and gestational hypertension (*P*<0.05), but not placental abruption and intrauterine fetal death.

**Table 3:** Correlation of serum vitamin D with maternal and infant outcomes

***Items***	***Cases***	***Deficiency (case, %)***	***Inadequacy (case, %)***	***Sufficiency (case, %)***	***χ^2^***	***P value***
Preterm birth						
Yes	7963	2662	2696	2605	13.718	0.001
No	15137	4864	4953	5320		
Gestational diabetes mellitus						
Yes	1344	477	501	366	32.007	p<.001
No	21756	7049	7148	7559		
Pre-eclampsia						
Yes	2103	753	751	599	34.984	p<.001
No	20997	6773	6898	7326		
Gestational hypertension						
Yes	1876	644	653	579	10.747	0.005
No	21224	6882	6996	7346		
Placental abruption						
Yes	464	155	165	144	2.434	0.296
No	22636	7371	7484	7781		
Intrauterine fetal death						
Yes	207	74	75	58	3.665	0.160
No	22893	7452	7574	7867		

## Discussion

In this study, 23100 pregnant women were included, and 7526 females showed vitamin D deficiency, accounting for 32.58%, and 7649 females showed vitamin D inadequacy, accounting for 33.11%. Vitamin D deficiency during the second trimester of gestation (16 weeks) is common among non-manual workers in Shanghai, China according to IOM criteria (9). In Shanghai about 68.6% pregnant women were vitamin D deficient, and 21.9% were vitamin D inadequate (9). Those data suggest that vitamin D deficiency were significantly improved in pregnant women in Shanghai during the past 5 years. Latitude and season can significantly affect the production of vitamin D3 (11, 12). Areas greater than 35 degree latitude almost received no ultraviolet rays, which is important for the synthesis of vitamin D3. Shanghai is around 31 latitudes. However, high prevalence of vitamin D deficiency was observed, which is almost comparable to that of females in Taiyuan, China (35 degree latitude) (13). The high prevalence of vitamin D deficiency in Shanghai is possibly caused by factors other than high latitudes, such as air pollution, less physical activity and sedentary occupations (6).

Aging has been proven to be closely correlated with vitamin D deficiency among pregnant women. Vitamin D level in pregnant women younger than 20 years were significantly lower than those older than 20 years (14). Pregnant women younger than 20 years were not included in this study. However, our data showed that aging was closely correlated with vitamin D deficiency among pregnant women during the second trimester of gestation, and being older than 30 yr is an independent risk factor for this disease.

Vitamin D deficiency is significantly more common among population without college education (15), which is consistent with the finding in our study that education level below colleges was an independent risk factor for vitamin D deficiency in second trimester of gestation.

Milk is an important food resource of vitamin D, and maternal milk restriction during pregnancy can significantly reduce the intake of vitamin D (16). Consistently, our study also showed that not consume milk products daily can significantly increase the incidence of vitamin D deficiency. Conclusions on the effects of smoking, drinking, use of vitamin D supplement and ethnic backgrounds on vitamin D status are still controversial (13, 15).

In our study, Being Han Chinese or minor group people showed no significant effect on vitamin D status, possibly due to the their similar lifestyle shaped by this rapidly developing city. However, smoking, drinking and no vitamin D supplement were proven to be independent risk factors for vitamin D deficiency. It is known that vitamin D status in human body depends on body mass index (BMI) (7).

In our study, BMI before pregnancy >26 and body weight gain during pregnancy<40g/day were proved to be independent risk factors for vitamin D deficiency, further confirming the effects of BMI on vitamin D status. Vitamin D status affects not only health of mothers but also infant outcomes (17, 18). Maternal vitamin D deficiency may cause pro-inflammatory responses and increase oxidative reactions in mothers and fetus, leading to a serious of adverse event (19). Low maternal vitamin D levels in pregnancy may be associated with an increased risk of preeclampsia, gestational diabetes mellitus and preterm birth. In another study, insufficient vitamin D was at least partially responsible for the occurrence of gestational hypertension (20). Consistently, in this study, vitamin D was significantly correlated with the occurrence of preterm birth, gestational diabetes mellitus, pre-eclampsia and gestational hypertension.

Placental abruption and intrauterine fetal death are two rare adverse pregnancy outcomes (21, 22). In this study, no significantly correlations were found between vitamin D status and the occurrences of placental abruption and intrauterine fetal death, which is inconsistent with another study (23), possibly due to the different ethnic background, different grouping method and the small sample size used in that study (n=227).

## Conclusion

Vitamin D deficiency rate was significantly improved during past 5 years but still high among women in second trimester of pregnancy in Shanghai China. Vitamin D deficiency in second trimester of pregnancy was closely correlated with the occurrence of a serious of adverse maternal and infant outcomes, such as preterm birth, pre-eclampsia and gestational hypertension and gestational diabetes mellitus.

## Ethical considerations

Ethical issues (Including plagiarism, informed consent, misconduct, data fabrication and/or falsification, double publication and/or submission, redundancy, etc.) have been completely observed by the authors.
